# Public Opinion on Use of Race in Clinical Algorithms

**DOI:** 10.1001/jamainternmed.2025.6929

**Published:** 2025-12-22

**Authors:** James A. Diao, Rajiv Movva, Lingwei Cheng, Kowe Kadoma, Aashna Shah, Neil R. Powe, Kadija Ferryman, Arjun K. Manrai, Emma Pierson

**Affiliations:** 1 Department of Biomedical Informatics, Harvard Medical School, Boston, Massachusetts; 2 Department of Medicine, Brigham and Women’s Hospital, Boston, Massachusetts; 3 Department of Electrical Engineering and Computer Sciences, University of California, Berkeley; 4 Heinz College of Information Systems and Public Policy, Carnegie Mellon University, Pittsburgh, Pennsylvania; 5 Department of Information Science, Cornell University, Ithaca, New York; 6 University of California San Francisco School of Medicine, San Francisco; 7 Zuckerberg San Francisco General Hospital and Trauma Center, San Francisco, California; 8 Berman Institute of Bioethics, Johns Hopkins University, Baltimore, Maryland; 9 Department of Health Policy and Management, Bloomberg School of Public Health, Johns Hopkins University, Baltimore, Maryland; 10 UCSF/UC Berkeley Joint Program in Computational Precision Health, University of California, Berkeley

## Abstract

This survey study assesses public opinion and preferences among US adults regarding the use of race in clinical algorithms.

The use of race in clinical algorithms remains debated. However, there are no large-scale quantitative data on patient preferences, an important consideration in algorithm design. Accordingly, we conducted a nationally representative survey of US adults to address this gap.

## Methods

In September 2024, we invited adults (aged ≥18 years) to complete an online survey using YouGov, a top-ranked polling platform.^1^ Participants comprised a general US population sample and separate samples of self-reported Asian, Black, and Hispanic adults. Samples were weighted using propensity scores to account for sampling and nonresponse bias. Two-sided *P* < .05 was significant. Statistical analyses were performed with R, version 4.4.0 (R Project for Statistical Computing). The Cornell University Institutional Review Board deemed this deidentified survey research exempt from review; participants provided informed consent. The study adhered to the AAPOR reporting guideline. Survey questions and additional analytic details are provided in the eAppendix and eMethods in Supplement 1.

## Results

Of 2638 invitees, 1997 (75.7%) completed the survey; 247 were excluded using attention and quality checks. Of the remaining 1750 respondents, the median (IQR) age was 45 (31-61) years; 916 (52.3%) were female and 834 (47.7%) were male (Table).

**Table.** Characteristics of Survey Respondents by Race and Ethnicity^a^

Characteristic	Unweighted						Overall weighted
	Asian (n = 275)	Black (n = 377)	Hispanic (n = 269)	White (n = 730)	Other (n = 99)^b^	Overall (N = 1750)	
Oversampling factor	2.16	1.73	1.37	0.63	1.73	1.00	NA
Completion time, median (IQR), min	9.5 (6.3-16.3)	13.1 (7.7-22.0)	10.5 (6.7-18.2)	12.0 (7.9-18.8)	11.4 (8.3-18.2)	11.6 (7.5-19.2)	11.7 (7.7-19.0)
Comprehension questions correct	233 (84.7)	298 (79.0)	221 (82.2)	660 (90.4)	89 (89.9)	1501 (85.8)	1501 (87.4)
Sex							
Female	155 (56.4)	183 (48.5)	146 (54.3)	374 (51.2)	58 (58.6)	916 (52.3)	916 (51.5)
Male	120 (43.6)	194 (51.5)	123 (45.7)	356 (48.8)	41 (41.4)	834 (47.7)	834 (48.5)
Age, y							
Median (IQR)	43 (31-57)	40 (27-59)	41 (29-56)	52.5 (35-64)	46 (34.5-61.5)	45 (31-61)	47 (32-63)
18-44	148 (53.8)	213 (56.5)	156 (58.0)	288 (39.5)	47 (47.5)	852 (48.7)	852 (46.1)
45-64	87 (31.6)	106 (28.1)	88 (32.7)	264 (36.2)	30 (30.3)	575 (32.9)	575 (32.6)
65-74	32 (11.6)	48 (12.7)	21 (7.8)	118 (16.2)	16 (16.2)	235 (13.4)	235 (14.9)
≥75	8 (2.9)	10 (2.7)	4 (1.5)	60 (8.2)	6 (6.1)	88 (5.0)	88 (6.4)
Educational attainment							
Less than high school	8 (2.9)	16 (4.2)	24 (8.9)	20 (2.7)	3 (3.0)	71 (4.1)	71 (6.8)
High school	267 (97.1)	361 (95.8)	245 (91.1)	710 (97.3)	96 (97.0)	1679 (95.9)	1679 (93.2)
4-y College	172 (62.5)	98 (26.0)	56 (20.8)	261 (35.8)	28 (28.3)	615 (35.1)	615 (34.3)
Family income, $							
0-79 999	112 (40.7)	272 (72.1)	190 (70.6)	400 (54.8)	79 (79.8)	1053 (60.2)	1053 (57.5)
≥80 000	121 (44.0)	65 (17.2)	53 (19.7)	258 (35.3)	10 (10.1)	507 (29.0)	507 (31.3)
Not reported	42 (15.3)	40 (10.6)	26 (9.7)	72 (9.9)	10 (10.1)	190 (10.9)	190 (11.2)
Employment status							
Full-time	119 (43.3)	119 (31.6)	112 (41.6)	291 (39.9)	33 (33.3)	674 (38.5)	674 (39.4)
Part-time	39 (14.2)	74 (19.6)	33 (12.3)	97 (13.3)	12 (12.1)	255 (14.6)	255 (12.9)
Not employed^c^	117 (42.5)	184 (48.8)	124 (46.1)	342 (46.8)	54 (54.5)	821 (46.9)	821 (47.7)
Political party							
Democrat	104 (37.8)	201 (53.3)	105 (39.0)	229 (31.4)	36 (36.4)	675 (38.6)	675 (35.5)
Republican	49 (17.8)	37 (9.8)	61 (22.7)	252 (34.5)	17 (17.2)	416 (23.8)	416 (27.4)
Neither	122 (44.4)	139 (36.9)	103 (38.3)	249 (34.1)	46 (46.5)	659 (37.7)	659 (37.1)
Urbanicity							
Urban	80 (29.1)	189 (50.1)	106 (39.4)	195 (26.7)	33 (33.3)	603 (34.5)	603 (31.4)
Suburban	163 (59.3)	121 (32.1)	119 (44.2)	293 (40.1)	33 (33.3)	729 (41.7)	729 (41.2)
Rural	21 (7.6)	33 (8.8)	31 (11.5)	159 (21.8)	22 (22.2)	266 (15.2)	266 (18.0)
Not reported	11 (4.0)	34 (9.0)	13 (4.8)	83 (11.4)	11 (11.1)	152 (8.7)	152 (9.4)
Self-rated health							
Fair or poor	39 (14.2)	41 (10.9)	44 (16.4)	76 (10.4)	20 (20.2)	220 (12.6)	220 (12.1)
Good	72 (26.2)	79 (21.0)	68 (25.3)	148 (20.3)	23 (23.2)	390 (22.3)	390 (22.3)
Very good or excellent	120 (43.6)	116 (30.8)	86 (32.0)	195 (26.7)	26 (26.3)	543 (31.0)	543 (28.7)
Not reported	44 (16.0)	141 (37.4)	71 (26.4)	311 (42.6)	30 (30.3)	597 (34.1)	597 (36.9)
Health care experiences							
Told that their race has been used in medical decisions	30 (10.9)	67 (17.8)	30 (11.2)	40 (5.5)	10 (10.1)	177 (10.1)	177 (8.6)
Believe that their race has been used in medical decisions	40 (14.5)	93 (24.7)	40 (14.9)	108 (14.8)	16 (16.2)	297 (17.0)	297 (16.0)
Mostly or always asked about race in health care settings	99 (36.0)	140 (37.1)	94 (34.9)	286 (39.2)	41 (41.4)	660 (37.7)	660 (37.9)
Believe that having a physician of the same race or ethnicity affects quality of care	97 (35.3)	150 (39.8)	89 (33.1)	175 (24.0)	29 (29.3)	540 (30.9)	540 (27.9)

Abbreviation: NA, not applicable.

^a^ Data are presented as No. (%) of participants unless indicated otherwise. Percentages are unweighted unless indicated otherwise.

^b^ Includes American Indian or Alaska Native (n = 17), Middle Eastern (n = 3), multiracial (n = 54), and other race (n = 25).

^c^ Includes student, homemaker, retired, disabled, unemployed, and other.

Most respondents were comfortable with the use of race in some circumstances. After reweighting, 79.8% (95% CI, 77.4%-82.1%) of adults preferred that their physician use all available information, including race, to make health decisions, whereas 20.1% (95% CI, 17.9%-22.6%) preferred that race be excluded (Figure, A). Similarly, 49.7% (95% CI, 46.8%-52.5%) of adults disagreed with the statement “There are no circumstances under which a doctor should use my race in clinical care,” whereas 22.0% (95% CI, 19.6%-24.4%) agreed (Figure, B). Preferences varied by race and ethnicity. For example, more Black (28.8% [95% CI, 23.7%-33.8%]) and Hispanic (30.4% [95% CI, 24.3%-36.5%]) respondents preferred that physicians exclude information on race compared with Asian (17.2% [95% CI, 12.1%-22.3%]) and White (17.1% [95% CI, 14.1%-20.1%]) respondents. When asked about a hypothetical cancer risk prediction algorithm, more respondents were uncomfortable with the use of income (39.3% [95% CI, 36.5%-42.1%]) and zip code (22.3% [95% CI, 19.9%-24.7%]) compared with race (8.8% [95% CI, 7.2%-10.5%]); fewer respondents were uncomfortable with the use of age or sex (Figure, C). Fewer respondents were uncomfortable with the use of race if physicians discussed how and why they were using it (8.3% [95% CI, 6.7%-9.9%]) vs using this information without asking (31.3% [95% CI, 28.6%-34.0%]) (Figure, D). However, few (8.6% [95% CI, 7.0%-10.2%]) reported ever being told that their race was used to make medical recommendations or decisions.

**Figure.**
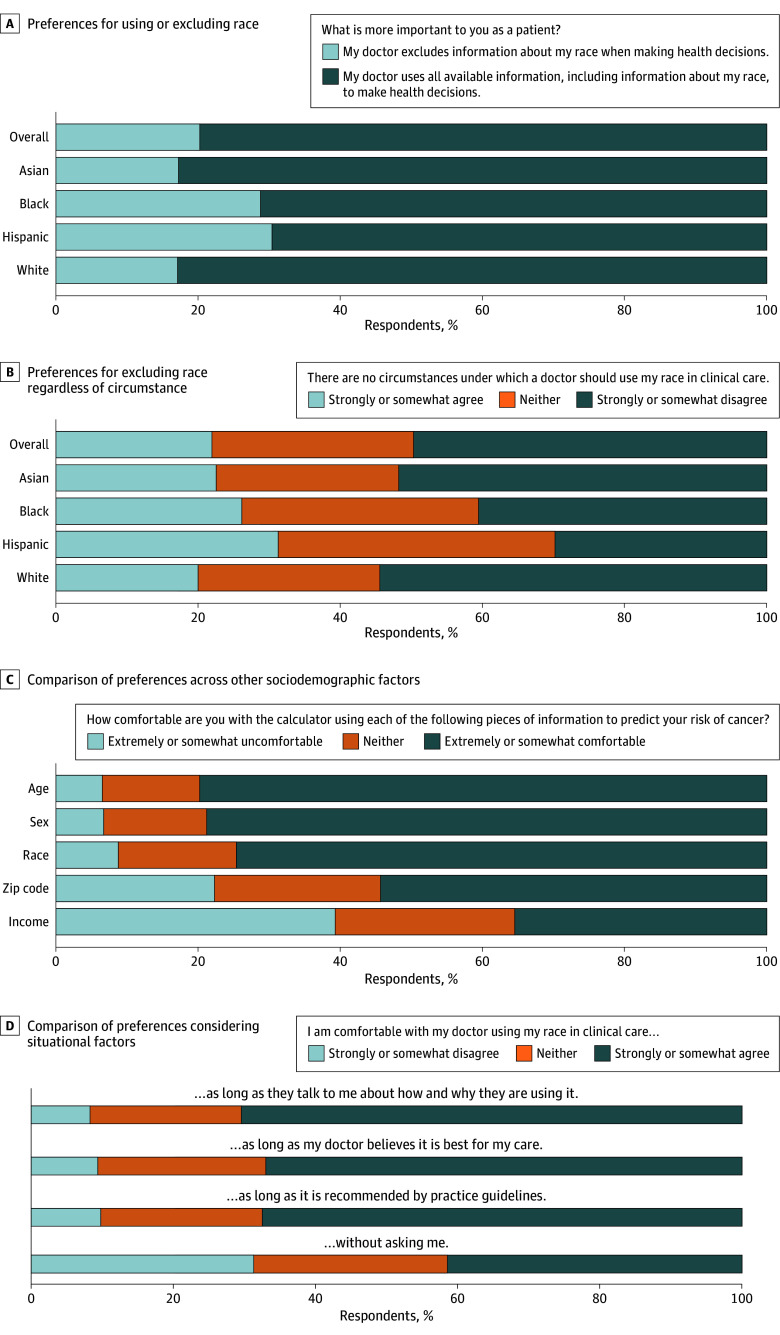
Survey Respondent Preferences on Use of Race in Clinical Algorithms A, Preferences for using or excluding race. B, Preferences for excluding race regardless of circumstance. C, Comparison of preferences across other sociodemographic variables. D, Consideration of situational factors. Response percentages were calculated using survey weights to account for selection and nonresponse bias. Direct quotes from the survey are presented here; complete survey questions are provided in the eAppendix in Supplement 1.

## Discussion

Our nationally representative survey study had 4 key findings. First, most individuals preferred that physicians use all available information, including race, to inform health decisions. This finding highlights a tension between calls to eliminate uses of race in medicine and attitudes or understandings among the public, whose support may be needed for a lasting resolution. Second, discomfort with the use of race varied across groups, with Black and Hispanic respondents more often opposed than Asian and White respondents. Differing opinions may reflect lived experiences that vary by race, ethnicity, and other social factors.^2^ Third, respondents were 4 times more likely to report discomfort when clinicians used race without asking. However, few respondents reported having ever been told that their race was used, despite earlier calls for transparency.^3^ Fourth, respondents were less comfortable with using income or zip code than with using race, suggesting that replacement of race with other social determinants of health may not be seen as more trustworthy.^4^

This study has limitations. We only included US adults who use the internet (96%),^5^ understand English well (92%),^6^ and consented to participate in a standing survey panel. Responses to hypothetical questions may not align with preferences in clinical settings. Finally, patient preferences are not sufficient to justify the inclusion or exclusion of race and should be considered alongside other criteria, including accuracy, equity, and outcomes.

## Notes

Supplement 1.
eMethods

eReferences
**eAppendix.** Survey Questions

Supplement 2.
Data Sharing Statement
