# Supplementary material for: Public Opinion on Use of Race in Clinical Algorithms
Source: JAMA Intern Med. 2025 Dec 22;186(2):266–9. doi: 10.1001/jamainternmed.2025.6929 (PMC12723594; doi:10.1001/jamainternmed.2025.6929)
Supplement: Supplement 1. — eMethods eReferences eAppendix. Survey Questions [file jamainternmed-e256929-s001.pdf]

Supplemental Online Content

Diao JA, Movva R, Cheng L, et al. Public opinion on use of race and ethnicity in clinical algorithms. *JAMA Intern Med*. Published online December 22, 2025.
doi:10.1001/jamainternmed.2025.6929

eMethods

eReferences

eAppendix. Survey Questions

This supplemental material has been provided by the authors to give readers additional information about their work.

© 2025 Diao JA et al. *JAMA Internal Medicine*.

eMethods

Survey Adjustment

Survey weights were produced from data including the 2020 American Community Survey,[1] 2020 Current Population Survey,[2] and the 2020 Cooperative Election Study.[3] Propensity scores were constructed based on age, gender, race/ethnicity, region, and years of education. Race/ethnicity was self-identified between the following options: Asian, Black, Hispanic, Middle Eastern, Native American, White, Two or more races, or Other. Participants answering "Two or more races" were then prompted to select from multiple race categories. Ethnicity was self-identified as Hispanic or Non-Hispanic.

Test of Differences in Proportion

We used the Rao-Scott likelihood ratio method to correct for survey design effects when performing the chi-squared test for difference in proportions between respondent groups.

Replication Questions

We compared responses to four replication questions on bias and AI in healthcare derived from the Pew Research Center (Pew)[1] and Kaiser Family Foundation (KFF)[2]. Questions included: (1) Same-race provider (KFF): "In general, do you think you would receive better or worse care from doctors or health care providers who share your racial and ethnic background, or would it not make much difference?"; (2) Comfort with AI in health care (Pew): "How would you feel if your health care provider relied on AI to do things like diagnose disease and recommend treatments for your medical care?"; (3) Bias in health care (Pew): "Thinking about the potential for bias and unfair treatment in health and medicine based on a patient's race or ethnicity, if artificial intelligence (AI) is used more in health and medicine to do things like diagnose disease and recommend treatments, do you think the issue of bias and unfair treatment based on a patient's race or ethnicity would definitely get better, probably get better, stay about the same,

© 2025 Diao JA et al. *JAMA Internal Medicine*.

probably get worse, definitely get worse, or no answer?" and (4) Bias if AI is used in health care (Pew): "In health and medicine, how much of a problem is bias and unfair treatment based on patients' race or ethnicity?" For all four replication questions, response proportions in our survey data (after reweighting) closely matched prior results from Pew and KFF.

Data and code availability

Survey data and code to replicate our results are available at https://github.com/epierson9/race_in_clinical_algorithms.

IRB

The Cornell Institutional Review Board determined that this research was exempt from review (IRB #0148620).

eReferences

1. Tyson A, Pasquini G, Spencer A, Funk C. 60% of Americans Would Be Uncomfortable With Provider Relying on AI in Their Own Health Care. February 22, 2023. Accessed February 18, 2025. https://www.pewresearch.org/wp-content/uploads/sites/20/2023/02/PS_2023.02.22_AI-health_REPORT.pdf
2. Samantha Artiga, Liz Hamel, Ana Gonzalez-Barrera, Alex Montero, Latoya Hill, Marley Presiado, Ashley Kirzinger, and Lunna Lopes. KFF Survey on Racism, Discrimination and Health. Kaiser Family Foundation. December 5, 2023. Accessed February 18, 2025. https://files.kff.org/attachment/Topline-KFF-Survey-on-Racism-Discrimination-and-Health.pdf

© 2025 Diao JA et al. *JAMA Internal Medicine*.

eAppendix. Survey Questions

Page: implicit_page_1

Page: implicit_page_race

race- Show if not pdl.race or pdl.race.last > months(12) or check_flag == 1/required SINGLE CHOICE

What racial or ethnic group best describes you?

varlabel Race - US

1 ○ White
2 ○ Black or African-American
3 ○ Hispanic or Latino
4 ○ Asian or Asian-American
5 ○ Native American
8 ○ Middle Eastern
6 ○ Two or more races
7 ○ Other (open [race_other])
98 *Skipped*
99 *Not Asked*

Page: p_hispanic if race != 3

hispanic- Show if not pdl.hispanic or pdl.hispanic.last >= months(12) or check_flag == 1/required SINGLE CHOICE

Are you of Spanish, Latino, or Hispanic origin or descent?

varlabel Hispanic - US

1 ○ Yes
2 ○ No
8 *Skipped*
9 *Not Asked*

Page: implicit_page_multrace

multrace- Show if race == 6 and (not pdl.multrace or pdl.multrace.last > months(12))/required MULTIPLE CHOICE

Please indicate the racial or ethnic groups that best describe you. (select all that apply)

© 2025 Diao JA et al. *JAMA Internal Medicine*.

varlabel	mixed race	

1 □ White
2 □ Black or African-American
3 □ Hispanic or Latino
4 □ Asian or Asian-American
5 □ Native American
8 □ Middle Eastern
97 □ Other — *Show if 0*
98 □ Don't know — *Exclude other punches*
99 □ None of these — *Show if 0*

Page: implicit_page_birthyr

birthyr- ***Show if not pdl.birthyr or int(pdl.birthyr) < 1920 or pdl.birthyr.last > months(27)/required*** — OPEN INTEGER TEXTBOX

In what year were you born?

cols	4
min	1900
varlabel	Birth Year
max	2024

Page: implicit_page_gender4

gender4- ***Show if (not pdl.gender4 or pdl.gender4.last > months(27))/required*** — SINGLE CHOICE

What is your gender?

varlabel	Gender 4-cat

1 ○ Man
2 ○ Woman
3 ○ Non-binary — *Not randomized*
4 ○ Other (open [gender4_t]) — *Not randomized*
8 *Skipped*
9 *Not Asked*

© 2025 Diao JA et al. *JAMA Internal Medicine*.

Page: implicit_page_educ

**educ- *Show if not pdl.educ or pdl.educ.last > months(12)/required***	SINGLE CHOICE
What is the highest level of education you have completed?	

varlabel Education

1 ○ Did not graduate from high school
2 ○ High school graduate
3 ○ Some college, but no degree (yet)
4 ○ 2-year college degree
5 ○ 4-year college degree
6 ○ Postgraduate degree (MA, MBA, MD, JD, PhD, etc.)
8 *Skipped*
9 *Not Asked*

Module: implicit_module_1

Page: implicit_page_2

Exit (quotafull) if not panman.qualified

Page: implicit_page_CRNL_races

**CRNL_races- *Show if 0/prompt once on skip***	SINGLE CHOICE
CRNL_races (calculated per client assignment logic)	

varlabel CRNL_races

1 ○ White
2 ○ Black or African American
3 ○ Hispanic
4 ○ Asian
8 *Skipped*
9 *Not Asked*

Page: picker2 if testing

This page is only shown during testing. Respondents will be randomly assigned.

© 2025 Diao JA et al. *JAMA Internal Medicine*.

demo_beyond_race_treatment- *prompt once on skip* SINGLE CHOICE

Demo beyond race treatment

varlabel Demo beyond race treatment

1 ○ With risk calculator accurate text
2 ○ Without risk calculator accurate text
8 *Skipped*
9 *Not Asked*

kidney_function_treatment- *prompt once on skip* SINGLE CHOICE

Kidney function treatment (only displayed to participants whose race/ethnicity is Black or African American)

varlabel Kidney function treatment

1 ○ Kidney transplant
2 ○ Kidney donation
8 *Skipped*
9 *Not Asked*

lung_emp_comp_questions_treatment- *prompt once on skip* SINGLE CHOICE

Lung emp comp situation treatment (only displayed to White/Hispanic/Asian/Black participants)

varlabel Lung emp comp situation treatment

1 ○ the lung-emp & hypo-lung-emp questions
2 ○ lung-comp & hypo-lung-comp questions
8 *Skipped*
9 *Not Asked*

cancer_screening_calculator_treatment- *prompt once on skip* SINGLE CHOICE

© 2025 Diao JA et al. *JAMA Internal Medicine*.

Calculator (uses race and other factors) predict cancer risk treatment

varlabel Calculator predict cancer risk treatment

1 ○ Calculator (no additional details)
2 ○ Calculator slightly more accurate than one that doesn't use race
3 ○ Calculator uncertain if more accurate
4 ○ Calculator controversial
8 *Skipped*
9 *Not Asked*

cancer_risk_prediction_treatment- *prompt once on skip* SINGLE CHOICE

Cancer risk prediction treatment

varlabel Cancer risk prediction treatment

1 ○ Higher risk
2 ○ Lower risk
8 *Skipped*
9 *Not Asked*

Page: implicit_page_3

Page: implicit_page_4

Page: intro_text_pg

Doctors use calculators to make predictions about patients' health risks, measure patient health, and make decisions about patient care. These calculators are also referred to as "algorithms" or, in some cases, "artificial intelligence (AI) models".

For example, one such calculator estimates breast cancer risk. It considers factors like age, race, family history of breast cancer, age when the patient had their first child, and other variables. The doctor can then use this risk estimate to determine if the patient needs additional breast cancer screening.

In this survey, we will ask you about what factors you are comfortable with doctors using to make these calculations about your own care (e.g., your race, age, or sex).

© 2025 Diao JA et al. *JAMA Internal Medicine*.

Please answer based on what you would want for yourself as a patient. Keep in mind that your answers may be shared anonymously with other researchers, so please do not share any information you are not comfortable sharing.

comprehension_check- *prompt once on skip* SINGLE CHOICE

Based on the information above, which of the following is a correct description of the breast cancer risk calculator?

varlabel	comprehension-check
order	randomize

1 ○ It uses information like the patient's age and race to make predictions about breast cancer risk.
2 ○ It can perfectly predict whether someone will get breast cancer.
3 ○ It does not use the patient's family history of breast cancer.
4 ○ It is never used by doctors.
8 *Skipped*
9 *Not Asked*

Page: implicit_page_medical_AI_literacy

medical_AI_literacy- *prompt once on skip* SINGLE CHOICE

As we described in this survey, doctors use simple calculators to make predictions about patients' health risks, measure patient health, and make decisions about patient care. How familiar are you with such calculators?

varlabel	medical-AI-literacy
order	reverse($rev_seed)

1 ○ Not at all familiar
2 ○ Slightly familiar
3 ○ Somewhat familiar
4 ○ Moderately familiar
5 ○ Extremely familiar
8 *Skipped*
9 *Not Asked*

Page: demo_beyond_race_pg

demo_beyond_race_grid- *prompt once on skip* GRID

Imagine a doctor is using a risk calculator to predict your risk of cancer. How comfortable are you with the calculator using each of the following pieces of information to predict your risk of cancer$demo_beyond_race_treatment_txt?

© 2025 Diao JA et al. *JAMA Internal Medicine*.

roworder	randomize
varlabel	demo-beyond-race
colorder	reverse($rev_seed)

ROWS

demo_beyond_race1- *prompt once on skip*	Your age
demo_beyond_race2- *prompt once on skip*	Your sex
demo_beyond_race3- *prompt once on skip*	Your race
demo_beyond_race4- *prompt once on skip*	Your income
demo_beyond_race5- *prompt once on skip*	Your home zipcode

COLUMNS

1 ○ Extremely uncomfortable
2 ○ Somewhat uncomfortable
3 ○ Neither comfortable nor uncomfortable
4 ○ Somewhat comfortable
5 ○ Extremely comfortable
8 *Skipped*
9 *Not Asked*

demo_justification OPEN TEXTBOX

Why or why not would you want the calculator to use the pieces of information above?

cols	40
rows	4
varlabel	demo-justification

Page: implicit_page_past_experience

past_experience- *prompt once on skip* SINGLE CHOICE

Has a doctor or nurse ever told you they were making a medical recommendation or decision in part because of your race?

© 2025 Diao JA et al. *JAMA Internal Medicine*.

varlabel past-experience

1 ○ Yes
2 ○ No
3 ○ Not sure
8 *Skipped*
9 *Not Asked*

past_experience_resp OPEN TEXTBOX

Can you provide more detail about a time when a doctor or nurse told you they were making a medical recommendation or decision based on your race?

cols	40
rows	4
varlabel	past-experience-resp

Page: implicit_page_believe_use_race

believe_use_race- ***Show if past_experience not in [1]/prompt once on skip*** SINGLE CHOICE

Regardless of whether they told you or not, do you believe doctors or nurses have made medical recommendations or decisions in part because of your race?

varlabel believe-use-race

1 ○ Yes
2 ○ No
3 ○ Not sure
8 *Skipped*
9 *Not Asked*

believe_use_race_res OPEN TEXTBOX

Can you provide more detail about a time when you believe a medical recommendation or decision was made based on your race, even though the doctor or nurse did not say so?

cols	40
rows	4

© 2025 Diao JA et al. *JAMA Internal Medicine*.

varlabel believe-use-race-res

Page: implicit_page_relevant_info

relevant_info- *prompt once on skip* SINGLE CHOICE

What is more important to you as a patient?

varlabel relevant-info

1 ○ My doctor uses all available information, including information about my race, to make health decisions.
2 ○ My doctor excludes information about my race when making health decisions.
8 *Skipped*
9 *Not Asked*

use_race_any OPEN TEXTBOX

Under what circumstances, if any, would you be comfortable with your doctor making different decisions for you on the basis of your race?

cols 40

rows 4

varlabel use-race-any

Page: implicit_page_bias_in_healthcare

bias_in_healthcare- *prompt once on skip* SINGLE CHOICE

In health and medicine, how much of a problem is bias and unfair treatment based on patients' race or ethnicity?

varlabel Bias-in-healthcare-pew-medbias

1 ○ A major problem
2 ○ A minor problem
3 ○ Not a problem
8 *Skipped*
9 *Not Asked*

© 2025 Diao JA et al. *JAMA Internal Medicine*.

Page: implicit_page_AI_in_healthcare

AI_in_healthcare- *prompt once on skip* SINGLE CHOICE

Thinking about the use of artificial intelligence (AI) in health and medicine to do things like diagnose disease and recommend treatment, how would you feel if your health care provider relied on AI to do things like diagnose disease and recommend treatments for your medical care?

varlabel	AI-in-healthcare-pew-aihccomf
order	reverse($rev_seed)

1 ○ Very comfortable
2 ○ Somewhat comfortable
3 ○ Somewhat uncomfortable
4 ○ Very uncomfortable
8 *Skipped*
9 *Not Asked*

Page: implicit_page_AI_bias_healthcare

AI_bias_healthcare- *prompt once on skip* SINGLE CHOICE

Thinking about the potential for bias and unfair treatment in health and medicine based on a patient's race or ethnicity, if artificial intelligence (AI) is used more in health and medicine to do things like diagnose disease and recommend treatments, do you think the issue of bias and unfair treatment based on a patient's race or ethnicity would:

varlabel	AI-bias-healthcare-pew-hcmedbias
order	reverse($rev_seed)

1 ○ Definitely get better
2 ○ Probably get better
3 ○ Stay about the same
4 ○ Probably get worse
5 ○ Definitely get worse
8 *Skipped*
9 *Not Asked*

Page: implicit_page_asked_race

asked_race- *prompt once on skip* SINGLE CHOICE

© 2025 Diao JA et al. *JAMA Internal Medicine*.

When you are seeking healthcare, how often are you asked what race group you belong to (either by the doctor or on a form)?

varlabel asked-race

order reverse($rev_seed)

1 ○ Never
2 ○ Sometimes
3 ○ About half the time
4 ○ Most of the time
5 ○ Always
6 ○ I am not sure *Not randomized*
8 *Skipped*
9 *Not Asked*

Page: implicit_page_care_preference

care_preference- *prompt once on skip* SINGLE CHOICE

In general, do you think you would receive better or worse care from doctors or health care providers who share your racial and ethnic background, or would it not make much difference?

varlabel care-preference-race-kff24

1 ○ Would receive better care
2 ○ Would receive worse care
3 ○ Wouldn't make much difference
8 *Skipped*
9 *Not Asked*

Page: implicit_page_statements_grid

statements_grid- *prompt once on skip* GRID

To what extent do you agree or disagree with the following statements?

roworder randomize

varlabel statements

colorder reverse($rev_seed)

ROWS

© 2025 Diao JA et al. *JAMA Internal Medicine*.

statements1- *prompt once on skip*	I believe perceptions of my race have negatively impacted my care.
statements2- *prompt once on skip*	My doctor should use my race in clinical care as long as my doctor believes it is best for my care.
statements3- *prompt once on skip*	I am comfortable with my doctor using my race in clinical care without asking me.
statements4- *prompt once on skip*	I am comfortable with my doctor using my race in clinical care if they talk to me about how and why they are using it.
statements5- *prompt once on skip*	I am comfortable with my doctor using my race in clinical care as long as it is recommended by practice guidelines.
statements6- *prompt once on skip*	There are no circumstances under which a doctor should use my race in clinical care.

COLUMNS

1 ○ Strongly disagree
2 ○ Somewhat disagree
3 ○ Neither agree nor disagree
4 ○ Somewhat agree
5 ○ Strongly agree
8 *Skipped*
9 *Not Asked*

Page: implicit_page_kidney_transplant

kidney_transplant- *Show if CRNL_races == 2 and kidney_function_treatment==1/prompt once on skip* SINGLE CHOICE

Imagine that your kidney health has been declining for years and your doctor thinks you would benefit from a kidney transplant. Only patients with kidney function scores below 20 are eligible. Your doctor asks if you want her to use your race when computing your score.She says that specific kidney function scores have been developed for Black patients. The score which accounts for your race will be higher, and so is more likely to say you are above the cutoff and not eligible for a kidney transplant. The score which does not account for your race will be lower, and so is more likely to say you are below the cutoff. As such, it will make you more likely to be eligible for a kidney transplant. Researchers have found that the score which accounts for your race can calculate kidney function slightly more accurately.Would you like the doctor to use your race when computing your score?

varlabel	kidney_transplant
order	reverse($rev_seed)

© 2025 Diao JA et al. *JAMA Internal Medicine*.

1 ○ I strongly prefer the score which uses race
2 ○ I slightly prefer the score which uses race
3 ○ I have no preference between the two scores
4 ○ I slightly prefer the score which does not use race
5 ○ I strongly prefer the score which does not use race
8 *Skipped*
9 *Not Asked*

Page: implicit_page_kidney_donation

kidney_donation- ***Show if CRNL_races == 2 and kidney_function_treatment==2/prompt once on skip*** SINGLE CHOICE

Imagine that your loved one's kidney health has been declining for years. Your doctor thinks they would benefit from a kidney transplant, and you are delighted to find that you are a match. Only people with kidney function scores above 60 are eligible to donate their kidney. Before testing your kidney function, your doctor asks if you want to use your race when computing your score.She says that specific kidney function scores have been developed for Black patients. The score which accounts for your race will be higher, and so is more likely to say you are above the cutoff and eligible to donate a kidney. The score which does not account for your race will be lower, and so is more likely to say you are below the cutoff. As such, it will make you less likely to be able to donate a kidney. Researchers have found that the score which accounts for your race can calculate kidney function slightly more accurately.Would you like the doctor to use your race when computing your score?

varlabel	kidney_donation
order	reverse($rev_seed)

1 ○ I strongly prefer the score which uses race
2 ○ I slightly prefer the score which uses race
3 ○ I have no preference between the two scores
4 ○ I slightly prefer the score which does not use race
5 ○ I strongly prefer the score which does not use race
8 *Skipped*
9 *Not Asked*

end module: implicit_module_1

Module: lung_emp_mod if CRNL_races in [1,2,3,4]

Page: implicit_page_lung_emp

© 2025 Diao JA et al. *JAMA Internal Medicine*.

lung_emp- *Show if lung_emp_comp_questions_treatment == 1/prompt once on skip* SINGLE CHOICE

Imagine that you work in construction and your six-figure salary supports your single-income family. Because your job involves exposure to dust and fumes, your employer requests a medical evaluation that includes a lung function test. People with low lung function are not eligible for construction work. Before testing your lung function, your doctor asks if you want him to use your race when computing your score.He says that using race accounts for differences in lung size across groups. Accounting for your race will give you a $lung_emp_race_score_txt score, making it $lung_emp_race_job_txt likely you will be eligible for the job. Not accounting for your race will give you a $lung_emp_no_race_score_txt score, making it $lung_emp_no_race_job_txt likely you will be eligible for the job. Would you like the doctor to use your race when computing your score?

varlabel	lung-emp
order	reverse($rev_seed)

1 ○ I strongly prefer the score which uses race
2 ○ I slightly prefer the score which uses race
3 ○ I have no preference between the two scores
4 ○ I slightly prefer the score which does not use race
5 ○ I strongly prefer the score which does not use race
8 *Skipped*
9 *Not Asked*

Page: implicit_page_hypo_lung_emp

hypo_lung_emp- *Show if lung_emp_comp_questions_treatment == 1/prompt once on skip* SINGLE CHOICE

Now imagine the doctor says the opposite. Accounting for your race will give you a $hypolung_emp_race_score_txt score, making it $hypolung_emp_race_job_txt likely you will be eligible for the job. Not accounting for your race will give you a $hypolung_emp_no_race_score_txt score, making it $hypolung_emp_no_race_job_txt likely you will be eligible for the job. Would you like the doctor to use your race when computing your score?

varlabel	hypo-lung-emp
order	reverse($rev_seed)

1 ○ I strongly prefer the score which uses race
2 ○ I slightly prefer the score which uses race

© 2025 Diao JA et al. *JAMA Internal Medicine*.

3 ○ I have no preference between the two scores
4 ○ I slightly prefer the score which does not use race
5 ○ I strongly prefer the score which does not use race
8 *Skipped*
9 *Not Asked*

Page: implicit_page_lung_comp

**lung_comp-** ***Show if lung_emp_comp_questions_treatment == 2/prompt once on skip***	SINGLE CHOICE

Imagine that you worked in construction for twenty years. You are worried that long-term exposure to dust and fumes at work has injured your lungs and limited your ability to breathe, and you believe you are owed financial compensation for your disability. To determine how much you receive in monthly payments, your employer requests a medical evaluation that includes a lung function test. People with lower lung function are eligible for greater payments. Before testing your lung function, your doctor asks if you want him to use your race when computing your score. He says that using race accounts for differences in lung size across groups. Accounting for your race will give you a $lung_comp_score_paymt_txt_1 score, and so will tend to result in $lung_comp_score_paymt_txt_2 payments. Not accounting for your race will give you a $lung_comp_score_paymt_txt_2 score, and so will tend to result in $lung_comp_score_paymt_txt_1 payments.Would you like the doctor to use your race when computing your score?

varlabel	lung-comp
order	reverse($rev_seed)

1 ○ I strongly prefer the score which uses race
2 ○ I slightly prefer the score which uses race
3 ○ I have no preference between the two scores
4 ○ I slightly prefer the score which does not use race
5 ○ I strongly prefer the score which does not use race
8 *Skipped*
9 *Not Asked*

Page: implicit_page_hypo_lung_comp

© 2025 Diao JA et al. *JAMA Internal Medicine*.

hypo_lung_comp- *Show if lung_emp_comp_questions_treatment == 2/prompt once on skip* SINGLE CHOICE

Now imagine if the doctor says the opposite. Accounting for your race will give you a $hypolung_comp_score_paymt_txt_1 score, and so will tend to result in $hypolung_comp_score_paymt_txt_2 payments. Not accounting for your race will give you a $hypolung_comp_score_paymt_txt_2 score, and so will tend to result in $hypolung_comp_score_paymt_txt_1 payments. Would you like the doctor to use your race when computing your score?

varlabel	hypo-lung-comp
order	reverse($rev_seed)

1 ○ I strongly prefer the score which uses race
2 ○ I slightly prefer the score which uses race
3 ○ I have no preference between the two scores
4 ○ I slightly prefer the score which does not use race
5 ○ I strongly prefer the score which does not use race
8 *Skipped*
9 *Not Asked*

end module: lung_emp_mod if CRNL_races in [1,2,3,4]

Module: implicit_module_2

Page: implicit_page_cancer_screening

cancer_screening- *prompt once on skip* SINGLE CHOICE

Imagine you go to your doctor for your annual checkup and your doctor says, "I want to ask you a few questions to help predict your risk of cancer so we can determine if you should start cancer screening". Cancer screening can lead to early detection and treatment of cancer, potentially increasing survival rates; however, it can also result in false positives that cause unnecessary anxiety and medical interventions, so is not recommended for people at very low risk. You have a choice between two risk prediction calculators: (1) A calculator which uses factors like your age and family history of cancer to predict your cancer risk, but does not use your race.(2) A calculator which uses your race, in addition to factors like age and family history of cancer, to predict your cancer risk.$accuracy_statement_txtThe calculator which uses your race predicts that your cancer risk is $higher_lower_text than the calculator which does not use your race.$screening_statementWould you want to start cancer screening now?

varlabel	cancer screening

© 2025 Diao JA et al. *JAMA Internal Medicine*.

order reverse($rev_seed)

1 ○ I definitely want to start cancer screening now
2 ○ I probably want to start cancer screening now
3 ○ I am not sure if I want to start cancer screening now
4 ○ I probably do not want to start cancer screening now
5 ○ I definitely do not want to start cancer screening now
8 *Skipped*
9 *Not Asked*

anything_else- ***prompt once on skip*** OPEN TEXTBOX

Studies related to health can be nuanced, with details beyond what can be covered within a single survey. Is there anything else you want to share with us? For more information on using race to calculate health risks, please visit $external_link_debrief.raw.

varlabel Final comments

Page: implicit_page_mena

mena- ***Show if race != 8 and (not pdl.mena or pdl.mena.last >= months(24))/required*** SINGLE CHOICE

Are you of Middle Eastern or North African origin or descent?

varlabel Middle Eastern or North African

1 ○ Yes
2 ○ No
8 *Skipped*
9 *Not Asked*

Page: gender_setup_page

Page: ask_gender_page if not pdl.gender or pdl.gender.last > months(27)

gender- ***Show if not pdl.gender or pdl.gender.last > months(27)/prompt once on skip*** SINGLE CHOICE

© 2025 Diao JA et al. *JAMA Internal Medicine*.

Are you…?

varlabel Gender

1 ○ Male
2 ○ Female
8 *Skipped*
9 *Not Asked*

Page: demos2

marstat- *Show if not pdl.marstat or pdl.marstat.last > months(12)/prompt once on skip* SINGLE CHOICE

What is your marital status?

varlabel Marital Status

1 ○ Married
2 ○ Separated
3 ○ Divorced
4 ○ Widowed
5 ○ Never married
6 ○ Domestic / civil partnership
8 *Skipped*
9 *Not Asked*

parent- *Show if not pdl.parent or pdl.parent.last > months(12)/prompt once on skip* MULTIPLE CHOICE

Are you a parent or guardian? Please select all that apply.

varlabel Parent of children younger or older than 18

1 □ Yes, of at least one child younger than 18 years old
2 □ Yes, of at least one child 18 years old or older
97 □ No, I am neither a parent or guardian *Exclude other punches*

© 2025 Diao JA et al. *JAMA Internal Medicine*.

child18- ***Show if 0/prompt once on skip*** SINGLE CHOICE

Are you the parent or guardian of any children under the age of 18?

varlabel	Children under the age of 18

1 ○ Yes
2 ○ No
8 *Skipped*
9 *Not Asked*

Page: implicit_page_speakspanish

speakspanish- ***Show if not pdl.speakspanish and pdl.race == 3 or 3 in pdl.multrace/required*** SINGLE CHOICE

Do you speak Spanish on a regular basis?

varlabel	Do you speak Spanish
topic	.hidden

1 ○ I speak Spanish primarily
2 ○ I speak both Spanish and English equally
3 ○ I speak English primarily but can speak Spanish
4 ○ I can not speak Spanish
8 *Skipped*
9 *Not Asked*

Page: work

employ- ***Show if not pdl.employ or pdl.employ.last > months(1)/prompt once on skip*** SINGLE CHOICE

Which of the following best describes your current employment status?

varlabel	Employment Status

1 ○ Working full time now
2 ○ Working part time now
3 ○ Temporarily laid off
4 ○ Unemployed

© 2025 Diao JA et al. *JAMA Internal Medicine*.

5 ○ Retired
6 ○ Permanently disabled
7 ○ Taking care of home or family
8 ○ Student
9 ○ Other (open [employ_t]) *Not randomized*
98 *Skipped*
99 *Not Asked*

Page: demos5

famin*c_new- *Show if (not pdl.faminc_new) or (pdl.faminc_new.last > months(6))/prompt once on skip SINGLE CHOICE
Thinking back over the last year, what was your family's annual income?

varlabel Family income

1 ○ Less than $10,000
2 ○ $10,000 - $19,999
3 ○ $20,000 - $29,999
4 ○ $30,000 - $39,999
5 ○ $40,000 - $49,999
6 ○ $50,000 - $59,999
7 ○ $60,000 - $69,999
8 ○ $70,000 - $79,999
9 ○ $80,000 - $99,999
10 ○ $100,000 - $119,999
11 ○ $120,000 - $149,999
12 ○ $150,000 - $199,999
13 ○ $200,000 - $249,999
14 ○ $250,000 - $349,999
15 ○ $350,000 - $499,999
16 ○ $500,000 or more
97 ○ Prefer not to say
998 *Skipped*
999 *Not Asked*

ownhome- *Show if not pdl.ownhome or pdl.ownhome.last > months(3)/prompt once on skip* SINGLE CHOICE

© 2025 Diao JA et al. *JAMA Internal Medicine*.

Do you own your home or pay rent?

varlabel Home ownership

1 ○ Own
2 ○ Rent
3 ○ Other (open [ownhome_t])
8 *Skipped*
9 *Not Asked*

Page: inputstate_page

inputstate- ***Show if not pdl.inputstate or pdl.inputstate.last > months(12)/prompt once on skip*** DROPDOWN

In which state do you live?

varlabel State of Residence

1 ○ Alabama
2 ○ Alaska
4 ○ Arizona
5 ○ Arkansas
6 ○ California
8 ○ Colorado
9 ○ Connecticut
10 ○ Delaware
11 ○ District of Columbia
12 ○ Florida
13 ○ Georgia
15 ○ Hawaii
16 ○ Idaho
17 ○ Illinois
18 ○ Indiana
19 ○ Iowa
20 ○ Kansas
21 ○ Kentucky
22 ○ Louisiana
23 ○ Maine
24 ○ Maryland

© 2025 Diao JA et al. *JAMA Internal Medicine*.

25 ○ Massachusetts
26 ○ Michigan
27 ○ Minnesota
28 ○ Mississippi
29 ○ Missouri
30 ○ Montana
31 ○ Nebraska
32 ○ Nevada
33 ○ New Hampshire
34 ○ New Jersey
35 ○ New Mexico
36 ○ New York
37 ○ North Carolina
38 ○ North Dakota
39 ○ Ohio
40 ○ Oklahoma
41 ○ Oregon
42 ○ Pennsylvania
44 ○ Rhode Island
45 ○ South Carolina
46 ○ South Dakota
47 ○ Tennessee
48 ○ Texas
49 ○ Utah
50 ○ Vermont
51 ○ Virginia
53 ○ Washington
54 ○ West Virginia
55 ○ Wisconsin
56 ○ Wyoming
60 ○ American Samoa
64 ○ Federated States of Micronesia
66 ○ Guam
68 ○ Marshall Islands
69 ○ Northern Mariana Islands
70 ○ Palau
72 ○ Puerto Rico
74 ○ U.S. Minor Outlying Islands
78 ○ Virgin Islands
81 ○ Alberta

© 2025 Diao JA et al. *JAMA Internal Medicine*.

82 ○ British Columbia
83 ○ Manitoba
84 ○ New Brunswick
85 ○ Newfoundland
86 ○ Northwest Territories
87 ○ Nova Scotia
88 ○ Nunavut
89 ○ Ontario
90 ○ Prince Edward Island
91 ○ Quebec
92 ○ Saskatchewan
93 ○ Yukon Territory
99 ○ Not in the U.S or Canada
998 *Skipped*
999 *Not Asked*

region- ***Show if 0/prompt once on skip*** SINGLE CHOICE
In which census region do you live?

varlabel Region

1 ○ Northeast
2 ○ Midwest
3 ○ South
4 ○ West
8 *Skipped*
9 *Not Asked*

end module: core

Module: politics_religion

Page: page_pid3

pid3- ***Show if not pdl.pid3 or pdl.pid3.last > months(1)/prompt once on skip*** SINGLE CHOICE
Generally speaking, do you think of yourself as a ...?

varlabel 3 point party ID

1 ○ Democrat

© 2025 Diao JA et al. *JAMA Internal Medicine*.

2 ○ Republican
3 ○ Independent
4 ○ Other (open [pid3_t])
5 ○ Not sure
8 *Skipped*
9 *Not Asked*

Page: page_pid7

pid7- ***Show if (not pdl.pid7 or pdl.pid7.last > months(1)) and pid3/prompt once on skip*** SINGLE CHOICE
$pid7text

varlabel 7 point Party ID

**1**	○	Strong Democrat	*Show if pid3==1*
**2**	○	Not very strong Democrat	*Show if pid3==1*
**7**	○	Strong Republican	*Show if pid3==2*
**6**	○	Not very strong Republican	*Show if pid3==2*
**3**	○	The Democratic Party	*Show if pid3 in [3,4,5]*
**5**	○	The Republican Party	*Show if pid3 in [3,4,5]*
**4**	○	Neither	*Show if pid3 in [3,4,5]*
**8**	○	Not sure	*Show if pid3 in [3,4,5]*
**9**	○	Don't know	*Show if 0*
**98**		*Skipped*	
**99**		*Not Asked*	

Page: voteregpage if pdl.inputstate not in [38]

votereg- ***Show if not pdl.votereg/required*** SINGLE CHOICE
Are you registered to vote?

varlabel Voter Registration Status

1 ○ Yes
2 ○ No
3 ○ Don't know
8 *Skipped*
9 *Not Asked*

© 2025 Diao JA et al. *JAMA Internal Medicine*.

Page: votereginterludepage

Module: vote2020_mod

Page: implicit_page_turnout20post

**turnout20post-** ***Show if not pdl.turnout20post/prompt once on skip***	SINGLE CHOICE
Did you vote in the November 2020 general election?	

varlabel 2020 Turnout

1 ○ Yes
2 ○ No
8 *Skipped*
9 *Not Asked*

Page: implicit_page_presvote20post

**presvote20post-** ***Show if turnout20post in [1] and not pdl.presvote20post/prompt once on skip***	SINGLE CHOICE
Who did you vote for in the election for President in 2020?	

varlabel 2020 President Vote Post Election

order reverse

**1**	○	Joe Biden	
**2**	○	Donald Trump	
**3**	○	Jo Jorgensen	*Not randomized*
**4**	○	Howie Hawkins	*Not randomized*
**5**	○	Other (open [presvote20post_t])	*Not randomized*
**6**	○	Did not vote for President	*Not randomized*
**8**		*Skipped*	
**9**		*Not Asked*	

end module: vote2020_mod

Page: demos6

**ideo5-** ***Show if not pdl.ideo5 or pdl.ideo5.last > months(1)/prompt once on skip***	SINGLE CHOICE
In general, how would you describe your own political viewpoint?	

varlabel Ideology

© 2025 Diao JA et al. *JAMA Internal Medicine*.

order reverse

1 ○ Very liberal
2 ○ Liberal
3 ○ Moderate
4 ○ Conservative
5 ○ Very conservative
6 ○ Not sure *Not randomized*
8 *Skipped*
9 *Not Asked*

newsint- *Show if not pdl.newsint or pdl.newsint.last > months(1)/prompt once on skip* SINGLE CHOICE

Some people seem to follow what's going on in government and public affairs most of the time, whether there's an election going on or not. Others aren't that interested. Would you say you follow what's going on in government and public affairs ...

varlabel Political Interest

1 ○ Most of the time
2 ○ Some of the time
3 ○ Only now and then
4 ○ Hardly at all
7 ○ Don't know *Not randomized*
98 *Skipped*
99 *Not Asked*

presvote16post- *Show if not pdl.presvote16post and pdl.birthyr <= 1998/prompt once on skip* SINGLE CHOICE

Who did you vote for in the election for President in 2016?

varlabel 2016 President Vote Post Election

order randomize

1 ○ Hillary Clinton
2 ○ Donald Trump
3 ○ Gary Johnson *Not randomized*
4 ○ Jill Stein *Not randomized*
5 ○ Evan McMullin *Not randomized*
6 ○ Other (open [presvote16post_t]) *Not randomized*

© 2025 Diao JA et al. *JAMA Internal Medicine*.

7 ○ Did not vote for President *Not randomized*
98 *Skipped*
99 *Not Asked*

Module: religion

Page: implicit_page_pew_bornagain

pew_bornagain- ***Show if not pdl.pew_bornagain or pdl.pew_bornagain.last > months(12)/prompt once on skip*** SINGLE CHOICE

Would you describe yourself as a "born-again" or evangelical Christian, or not?

varlabel Born Again (Pew version)

1 ○ Yes
2 ○ No
8 *Skipped*
9 *Not Asked*

Page: implicit_page_pew_religimp

pew_religimp- ***Show if not pdl.pew_religimp or pdl.pew_religimp.last > months(12)/prompt once on skip*** SINGLE CHOICE

How important is religion in your life?

varlabel Importance of religion (Pew version)

1 ○ Very important
2 ○ Somewhat important
3 ○ Not too important
4 ○ Not at all important
8 *Skipped*
9 *Not Asked*

Page: implicit_page_pew_churatd

pew_churatd- ***Show if not pdl.pew_churatd or pdl.pew_churatd.last > months(12)/prompt once on skip*** SINGLE CHOICE

© 2025 Diao JA et al. *JAMA Internal Medicine*.

Aside from weddings and funerals, how often do you attend religious services?

varlabel Church attendance (Pew version)

1 ○ More than once a week
2 ○ Once a week
3 ○ Once or twice a month
4 ○ A few times a year
5 ○ Seldom
6 ○ Never
7 ○ Don't know *Not randomized*
98 *Skipped*
99 *Not Asked*

Page: implicit_page_pew_prayer

pew_prayer- *Show if not pdl.pew_prayer or pdl.pew_prayer.last > months(12)/prompt once on skip* SINGLE CHOICE

People practice their religion in different ways. Outside of attending religious services, how often do you pray?

varlabel Frequency of Prayer (Pew version)

1 ○ Several times a day
2 ○ Once a day
3 ○ A few times a week
4 ○ Once a week
5 ○ A few times a month
6 ○ Seldom
7 ○ Never
8 ○ Don't know
98 *Skipped*
99 *Not Asked*

Page: implicit_page_religpew

religpew- *Show if not pdl.religpew or pdl.religpew.last > months(12)/prompt once on skip* SINGLE CHOICE

What is your present religion, if any?

varlabel Religion

© 2025 Diao JA et al. *JAMA Internal Medicine*.

1 ○ Protestant
2 ○ Roman Catholic
3 ○ Mormon
4 ○ Eastern or Greek Orthodox
5 ○ Jewish
6 ○ Muslim
7 ○ Buddhist
8 ○ Hindu
9 ○ Atheist
10 ○ Agnostic
11 ○ Nothing in particular
12 ○ Something else (open [religpew_t])
98 *Skipped*
99 *Not Asked*

© 2025 Diao JA et al. *JAMA Internal Medicine*.
