# Supplementary material for: Public Opinion on Use of Race in Clinical Algorithms
Source: JAMA Intern Med. 2025 Dec 22;186(2):266–9. doi: 10.1001/jamainternmed.2025.6929 (PMC12723594; doi:10.1001/jamainternmed.2025.6929)
Supplement: Supplement 2. — Data Sharing Statement [file jamainternmed-e256929-s002.pdf]

Data Sharing Statement

Diao. Public Opinion on Use of Race in Clinical Algorithms. *JAMA Intern Med*. Published December 22, 2025. doi:10.1001/jamainternmed.2025.6929

Data
Data available: Yes
Data types: Deidentified participant data, Data dictionary
How to access data: We will release the data and the analysis code in a public Github repository (https://github.com/epierson9/race_in_clinical_algorithms).
When available: With publication

Supporting Documents
Document types: Statistical/analytic code
How to access documents: We will release the data and the analysis code in a public Github repository (https://github.com/epierson9/race_in_clinical_algorithms).
When available: With publication

Additional Information
Who can access the data: Anyone who would like the data will be able to download it from Github (https://github.com/epierson9/race_in_clinical_algorithms).
Types of analyses: Any purpose.
Mechanisms of data availability: Public.
Any additional restrictions: None.
